# Differences in lipidome and metabolome organization of prefrontal cortex among human populations

**DOI:** 10.1038/s41598-019-53762-6

**Published:** 2019-12-04

**Authors:** Anna Tkachev, Vita Stepanova, Lei Zhang, Ekaterina Khrameeva, Dmitry Zubkov, Patrick Giavalisco, Philipp Khaitovich

**Affiliations:** 1 Skolkovo Institute of Science and Technology, 121205 Moscow, Russia; 2 Institute for Information Transmission Problems, Russian Academy of Sciences, Bolshoy Karetny Per. 19/1, 127051 Moscow, Russia; 3 CAS Key Laboratory of Computational Biology, CAS-MPG Partner Institute for Computational Biology, 320 Yue Yang Road, 200031 Shanghai, China; 4 Max Planck Institute for Biology of Ageing, Joseph-Stelzmann-Straße 9B, 50931 Cologne, Germany; 5 Max Planck Institute for Evolutionary Anthropology, Deutscher Platz 6, 04103 Leipzig, Germany

**Keywords:** Molecular evolution, Molecular neuroscience

## Abstract

Human populations, despite their overwhelming similarity, contain some distinct phenotypic, genetic, epigenetic, and gene expression features. In this study, we explore population differences at yet another level of molecular phenotype: the abundance of non-polar and polar low molecular weight compounds, lipids and metabolites in the prefrontal cortical region of the brain. We assessed the abundance of 1,670 lipids and 258 metabolites in 146 Han Chinese, 97 Western European, and 60 African American individuals of varying ages, covering most of the lifespan. The statistical analysis and logistic regression models both demonstrated extensive lipid and metabolic divergence of the Han Chinese individuals from the other two populations. This divergence was age-dependent, peaking in young adults, and involved metabolites and lipids clustering in specific metabolic pathways.

## Introduction

There is ample evidence of genetic differences among populations, including neutral genetic divergence and examples of local adaptations^1–3^. Differences among populations were also shown at the levels of DNA methylation^4^, expression of protein-coding genes in placental tissue^5^, lymphoblastoid cell lines^6,7^, whole blood^8^, as well as expression of small non-coding RNA (microRNA)^9,10^.

Phenotypically, populations differ in disease susceptibility, including frequency of certain brain disorders. Specifically, African Americans show lower occurrence of Parkinson’s disease^11,12^ and amyotrophic lateral sclerosis^13^, and a higher occurrence trend for Alzheimer’s disease^14^. Moreover, population differences have been reported for episodic and semantic memory, as well as executive functioning^15,16^. Anatomically, population differences in cortical thickness values, as well as brain region volume and surface area, have been reported for multiple brain areas^17,18^.

Population differences in brain molecular organization were addressed in one study exploring mRNA and lipid abundance levels in the prefrontal cortex of the brain in 14 individuals of East Asian (*n* = 5), Western European (*n* = 5), and African American (*n* = 4) descent^19^. Despite limited sample size, the study indicated the presence of population differences at both mRNA and lipid abundance levels.

While lipids (organic compounds mostly insoluble in water, but soluble in non-polar organic solvents) constitute most of the brain’s dry mass and are crucial for membrane architecture and geometry, cell signaling, and protein anchoring^20^, lipidome organization of the human brain remains largely unexplored. At the same time, lipids contribute significantly to healthy neural development^21,22^, CNS disorder progression^23,24^, and response to medical treatment^25^. In addition to lipids, differences in the abundance of polar metabolites (organic compounds soluble in water) in the prefrontal cortex were linked to neuropsychiatric disorders - including schizophrenia and psychosis - and were also associated with cognitive abilities, such as memory and orientation^26–30^. Moreover, the lipid and metabolite composition of the human brain was demonstrated to undergo substantial age-dependent rearrangements, especially during early postnatal development^31–33^. These observations suggest that lipidome and metabolome composition of the human brain is an important component of the brain functionality, as well as a potentially important element determining brain disorder susceptibility and treatment efficiency.

Although the existence of population differences in brain lipidome composition was suggested by the prefrontal cortex study of 14 individuals from three populations^19^, its sample size prevented detailed analysis of these differences. Here, we conducted a more extended survey of brain composition variation among human populations by assessing the abundance differences among Han Chinese (HC), Western European (WE), and African American (AA) populations for 1,670 distinct lipid peaks and 258 polar water-soluble brain metabolites in a total of 303 individuals. In addition to covering more individuals, our study explored the dependence of the population-specific differences on individuals’ age and included polar metabolites, thus assessing a level of molecular brain organization not explored in previous studies.

## Results

### Lipidome and metabolome variation analysis

**Figure 1:**
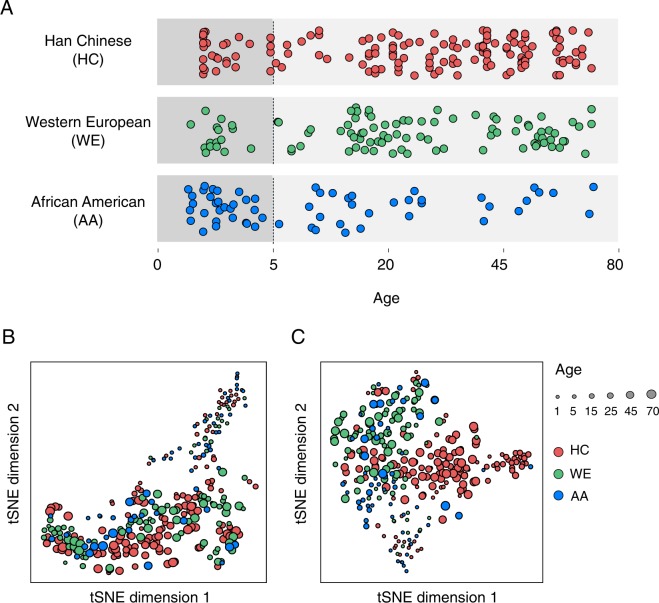
Assessment of the lipid and metabolite abundance variation. (**A**) Age distribution of samples. Each circle represents an individual. The circle colors correspond to populations: red – Han Chinese (HC), green – Western Europeans (WE), blue – African Americans (AA). The background color delineates two datasets: the darker shade – samples with ages less than 5 years (DS:0-4) and the lighter shade – samples with ages greater than 5 years (DS:5-71). (**B**,**C**) Lipid (**B**) and metabolite (**C**) abundance variation among individuals visualized using t-distributed stochastic neighbor embedding (t-SNE). Each circle represents an individual. The sizes of the circles are related to the individuals’ age – larger circles represent older individuals. Colors represent populations, as described above.

We assessed the abundance of lipids and polar metabolites in the prefrontal cortex samples of 146 Han Chinese (HC), 97 Western European (WE), and 60 African American (AA) individuals (Table S1). For each population, the ages of individuals covered most of the lifespan: from birth to 71 years of age (Fig. 1A). For each individual, the lipids and metabolites were extracted from the same cortical gray matter sample dissected from the dorsolateral region of the prefrontal cortex. Tissue preservation of the samples was assessed using postmortem interval duration (PMI) and RNA integrity number (RIN) measured for a subset of individuals (Table S1).

The lipid abundance measurements were conducted using liquid chromatography coupled with untargeted mass spectrometry (LC-MS) in positive and negative ionization modes. The LC-MS measurements yielded a total of 1,670 distinct lipid peaks not affected by the confounding factors, such as extraction batch, mass spectrometry loading order, and PMI (Table S2). Among them, 900 peaks were computationally annotated (Table S3) based on mass-to-charge ratio values using LIPID MAPS Structure Database (LMSD)^34^. All analyses of the lipidome population differences were based on the intensities of 1,670 detected lipids unless indicated otherwise.

The metabolite abundance measurements were conducted using gas chromatography coupled with mass spectrometry (GC–MS). The GC-MS measurements yielded 258 confounder-free compounds identified and annotated using previously analyzed metabolite standards (Table S4). All analyses of the metabolome population differences were based on the intensities of these 258 metabolites unless indicated otherwise.

The overall lipidome variation analysis conducted using t-distributed stochastic neighbor embedding (t-SNE) based on the abundance of 1,670 detected or 900 computationally annotated lipids showed strong separation of the youngest individuals from the rest (Figs 1B and S1). The same result was observed based on the abundance of 258 detected polar metabolites (Fig. 1C). Nonetheless, the trend distinguishing samples of different populations was also apparent in both the lipid and metabolite data. Correspondingly, the variation analysis of the lipid abundance levels indicated that age explained 28% of the total variation and population identity 3%. Other factors, such as sex, RIN, and PMI each explained less than 1% of the total lipidome variation. Similarly, for the polar compounds, population identity explained 6% of the total variation, and the other factors less than 2.5% each (Fig. S2).

### Statistical analysis of lipid and metabolite differences among populations

**Figure 2:**
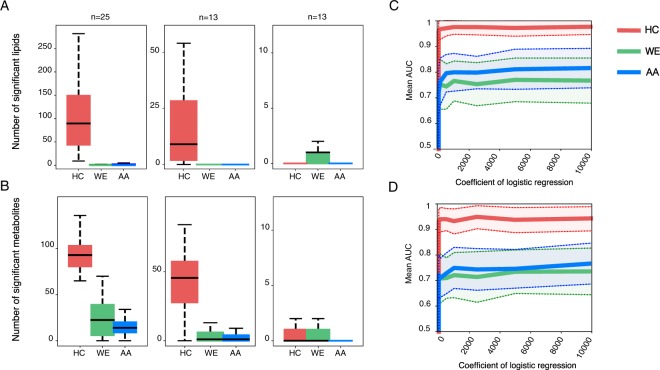
Lipidome and metabolome population-specific differences in the DS:0-4 and DS:5-71 datasets. (**A**,**B**) Number of lipids (**A**) and metabolites (**B**) with significant abundance differences between one population and the other two combined, estimated by subsampling n individuals from each of the three populations 100 times. The numbers of subsampled individuals n used in analysis are marked on top of the panels. The results are shown for DS:5-71 (left, n = 25), DS:5-71 with same number n as DS:0-4 (middle, n = 13), and DS:0-4 (right, n = 13). The colors represent populations: red – Han Chinese (HC), green – Western Europeans (WE), blue – African Americans (AA). (**C**,**D**) The mean Area Under the ROC Curve (AUC) estimates for the lasso logistic regression models separating samples from one population and samples from the other two combined calculated using different values of hyperparameter C (inverse of regularization strength). The models were based on the lipid (**C**) and metabolite (**D**) abundance in DS:5-71 samples. Lines correspond to the means of AUC values estimated on different test sets. The shaded areas indicate the standard deviations of AUC values estimated on different test sets. The colors correspond to populations, as described above.

Consistent with previous reports^32^, variation analysis indicated the presence of strong age-dependent lipidome and metabolome differences between samples of younger ages and the remaining individuals (Fig. 1B,C). To reduce the influence of age on the inter-populational variation, we separated the samples into two datasets: DS:0–4 (*n* = 74, ages less than five years, Table S1), and DS:5–71 (*n* = 229, ages from five to 71 years, Table S1) (Fig. 1A). We then searched for lipid abundance differences characteristic of each population by comparing it to the other two populations. To equalize the statistical power, we subsampled the same number of individuals per population in every comparison 100 times (*n* = 13 for DS:0–4 and *n* = 25 for DS:5–71) and identified lipids showing population-specific abundance levels in each subsampling. In DS:0–4, this analysis revealed no lipid abundance differences specific to any particular population, with a marginally higher number of differences specific to WE (median = 1 for WE, median = 0 for HC and AA, t-test, Benjamini-Hochberg corrected *p* < 0.05; Fig. 2A). By contrast, in DS:5–71, HC population differed from the other two by the median of 90 lipids, while no lipids showed abundance levels specific to WE and AA in the average of 100 sample subsets (t-test, Benjamini-Hochberg corrected *p* < 0.05; Fig. 2A). The specific lipidome behavior of HC population in DS:5–71 was not due to the difference in statistical power between DS:5–71 and DS:0–4, as shown by subsampling the same number of individuals in both datasets (Fig. 2A). Restricting the analysis to well-preserved DS:5–71 samples defined based on RNA conservation and an empirically determined RNA quality threshold^35^ (RIN ≥ 7, *n* = 82; Fig. S3; Table S1) retained an evident excess of the HC-specific lipid abundance differences, compared to AA and WE population-specific differences (Fig. S4A). Similarly, exclusion of lipids showing even weak correlation between the abundance and PMI duration (definition of PMI effect threshold relaxed to: absolute value of correlation coefficient *r* = 0.137, *p* = 0.1) did not alter the results (Fig. S5A; Table S5).

Analysis based on all DS:5–71 samples yielded 395 lipids showing abundance levels specific to HC population (t-test, Benjamini-Hochberg corrected *p* < 0.05; Table S6). Notably, the comparison to an independently generated published adult cortical lipidome dataset consisting of five HC, five WE, and four AA individuals^19^ confirmed the identified lipid concentration differences specific to HC population (Spearman correlation test, *p* = 0.003; Table S7).

The same statistical analysis applied to the polar metabolite dataset produced similar results. While there were no metabolite concentration differences specific to either of the three populations in DS:0–4 the median of 93 metabolites showed abundance levels particular to HC, 23 – to WE, and 14 – to AA in DS:5–71 (t-test, Benjamini-Hochberg corrected *p* < 0.05; Fig. 2B). Similar to the lipid data, the excess of metabolic differences particular to HC population in DS:5–71 remained robust after subsampling the same number of individuals in both DS:5-71 and DS:0-4 (Fig. 2B). Restriction of analysis to well preserved DS:5-71 samples (RIN ≥ 7, *n* = 80) or exclusion of polar metabolites showing even weak correlation between the abundance and PMI duration (definition of PMI effect threshold: absolute value of correlation coefficient *r* = 0.170, *p* = 0.1) retained an evident excess of the HC-specific metabolite abundance differences (Figs S4B, S5B; Table S8).

Analysis based on all DS:5-71 samples yielded 166 HC-specific metabolite differences (t-test, Benjamini-Hochberg corrected *p* < 0.05; Table S9).

### Population classification using machine learning

To test whether the populations could be distinguished reliably based on lipid or polar metabolite abundance values, we classified samples using lasso logistic regression model. Because of the strong effect of age on compound abundances resulting in the separation of samples from very young individuals (Fig. 1B,C), the classification procedure was applied to DS:5-71 only and not to the complete set of samples. The classification procedure was not applied to the DS:0-4 separately because of insufficient sample size.

For the lipid data, the resulting model accurately separated the HC population from the other two (area under the curve AUC = 0.97) (Figs 2C and S6A). The separation of AA population, as well as WE population, was notably less accurate (AUC = 0.8 and 0.76, respectively), although still significantly better than expected by chance (Figs 2C and S6A). We used stability selection procedure^36^ to define lipid predictors of HC population (Table S6). These predictors (*n* = 200) overlapped well with statistically defined lipid abundance differences (hypergeometric test, *p* = 3.5e^−54^; Fig. S7A; Table S6). Notably, the abundance differences of lipid predictors between HC and the other two populations correlated with the differences determined using an independent published dataset^19^ (Spearman correlation test, *p* = 0.005; Table S7). Furthermore, validation of the model on this independently generated published dataset resulted in good classification of HC individuals (AUC = 0.89; Fig. S8). It has to be noted that model accuracy estimates were limited in this case by the size of the published dataset.

Application of the logistic regression model to metabolite DS:5-71 dataset similarly resulted in significantly higher classification accuracy for HC individuals: AUC = 0.94 compared to AUC = 0.72 for WE, and AUC = 0.74 for AA (Figs 2D and S6B). Stability selection procedure^36^ yielded 50 metabolite predictors of HC population, which overlapped well with the statistically defined differences (hypergeometric test, *p* = 0.002; Fig. S7B; Table S9).

### Age dynamics of population differences

The statistical analysis of lipid and metabolite abundance yielded detectable HC-specific differences only in DS:5-71 samples. Similarly, the accuracy of the logistic regression model trained on DS:5-71 data remained mostly unchanged when applied to classification of AA and WE samples with ages from 0 to 4 years, but dropped drastically when applied to classification of HC samples from 0–4 years age interval (Fig. S9). Notably, this drop in accuracy was detected at approximately two years of age, even though all samples with age <5 years were excluded from the training set of the logistic regression model. These results suggest that the detected HC-specific differences cannot be generalized to HC individuals younger than two years of age.

**Figure 3:**
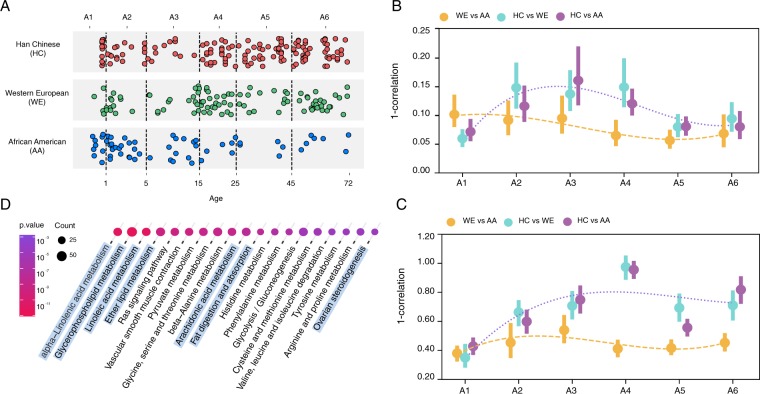
The age-dependent dynamics of lipidome and metabolome differences between populations. (**A**) Sample distribution across six age groups. Each circle represents an individual. The colors represent populations: red – Han Chinese (HC), green – Western Europeans (WE), blue – African Americans (AA). The x-axis labels indicate the age groups’ boundaries in years. (**B**,**C**) Pairwise population differences estimated based on the abundance of age-unbiased population-distinguishing lipids (**B**) and metabolites. (**C**) Differences were calculated in each age group A1-A6 using correlations of population-mean abundances based on four samples subsampled from each population. Y-axis represents distance values calculated as one minus these correlation values. Circles represent the median distance values estimated by subsampling within each age group 10,000 times. Vertical lines extend to the upper and lower quartile values in each age group. The dotted blue line represents a smooth spline curve fitted to the average of the HC-WE and HC-AA distances. The dashed orange line represents a smooth spline curve fitted to the WE-AA distance. (**D**) Pathway enrichment analysis. Shown are the top 19 pathways (Benjamini-Hochberg corrected hypergeometric test *p* < 0.001) that show enrichment of genes linked to HC-specific lipids and metabolites. HC-specific lipids and metabolites defined from stability selection were used in this analysis. Circle sizes represent the number of genes linked to HC-specific lipid and metabolite compounds. Circle colors correspond to Benjamini-Hochberg corrected hypergeometric test p-values. Pathways associated with lipid metabolism are shaded in light blue.

To assess the relationship between the population differences and individuals’ age, we divided all individuals into six age groups A1-A6, separated at 1, 5, 15, 25, 45 years of age and containing 24–75 individuals each (Fig. 3A; Table S1). We then subsampled the same number of individuals of each population within each age group (*n* = 4) 1,000 times and identified top 50 lipids or top 20 metabolites showing the most consistent abundance differences between each population pair in each age group (t-test, nominal *p* < 0.1). We used the union of these lipids or metabolites to construct a set of age-unbiased population-distinguishing compounds. The distances calculated from correlations of population-mean abundances of these compounds showed strong age-dependent behavior of HC-related differences for both lipids and metabolites (Fig. 3B,C). Specifically, the distances between HC and the other two populations increased substantially after the first year of life and then decreased after 20–30 years of age (Fig. 3B,C). Thus, age groups contributed unevenly to the separation of HC samples from the other two populations in the DS:5-71 sample set, with the strongest contribution provided by the young adult groups. This pattern was even more pronounced for HC-specific compound abundances (Fig. S10). By contrast, AA-WE distance did not show any substantial increase along the lifespan (Figs 3B,C and S10). Additionally, among all six age groups, the HC-AA and HC-WE distances were the smallest in A1 age group. This result is consistent with inaccurate logistic regression model performance for classification of HC individuals younger than two years of age (Fig. S9).

### Functional characterization of HC-specific lipid and metabolite differences

We assessed potential functions of lipids and polar metabolites distinguishing adult HC individuals by testing their enrichment in functional pathways defined by KEGG (Kyoto Encyclopedia of Genes and Genomes)^37^. The enrichment analysis involved 900 computationally annotated lipids (Table S3) and 258 polar annotated metabolites and was based on the comparison between genes linked to HC-specific compounds (Tables S6 and S9) and genes linked to the other detected compounds according to KEGG database. The analysis yielded a total of 35 significantly enriched pathways, including ten pathways associated with amino acid metabolism and seven pathways associated with lipid metabolism (hypergeometric test, Benjamini-Hochberg corrected *p* < 0.05; Table S10). Notably, all seven pathways associated with lipid metabolism were present in the top 19 enriched terms (hypergeometric test, Benjamini-Hochberg corrected *p* < 0.001; Fig. 3D).

## Discussion

While lipids constitute the majority of the human brain’s organic material and are essential for brain functionality, only a handful of studies to date examined human brain lipidome composition^19,21,31,32,38–47^. Among them, one study assessed lipid composition of the prefrontal cortex in 14 individuals representing three populations, Han Chinese (HC), Western European (WE), and African American (AA), suggesting possible lipid abundance differences among populations^19^. Our study expanded this work by including 303 individuals of different ages representing the same three populations. Our analysis indicates the robust presence of lipid and polar metabolite abundance differences distinguishing the prefrontal cortex composition of Han Chinese (HC) individuals from that of Western Europeans (WE) and African Americans (AA). The observed difference between HC individuals and the other two populations is age-dependent: it peaks at approximately 20 years of age and is absent during the first year of life. The difference was robust to the sample quality variation estimated using RIN and PMI values, as well as to within-population variability estimated by subsampling individuals within populations. Furthermore, reanalysis of the lipidome data from the previous study based on 14 individuals^19^ revealed HC-specific differences coinciding with the lipid abundance differences detected in our study.

The separation of adult HC individuals from WE and AA individuals with respect to lipid and polar metabolite abundance composition of the prefrontal cortex is a novel observation, which contrasts with genetic and gene expression distances reported among populations. Specifically, the three populations used in the analysis are approximately equidistant from one another at the genome level, given the admixed genetic background of AA individuals^48,49^. Similarly, no excess of HC- or east Asian-specific differences were reported by studies examining population-specific gene expression variation^5,10^. Nonetheless, some epigenetic effects, such as hypomethylation at *BRSK2*, were shown to be characteristic of East Asian individuals^50^. Furthermore, a concentration pattern specific to HC was shown at the lipidome level in a study analyzing whole blood composition in Chinese, Malay, and Indian individuals, although most lipid abundance differences were reported between individuals of Indian and non-Indian descent^8^.

The absence of pronounced lipid and metabolic differences distinguishing HC individuals during the first year of life, the period characterized by more uniform feeding and living routines, suggests that observed HC-specific differences might be environmental. On the other hand, studies examining dietary effects on different tissues, conducted in mice at the gene expression level^51^ and in macaques at the lipid abundance level^43^, reported little or no detectable dietary effects in the brain, in contrast to non-neural tissues. Furthermore, the clustering of HC-specific lipid and metabolite concentration differences in particular functional pathways, detected in our study, might imply a possible link between these differences and variation in brain organization, functionality, and disease susceptibility among human populations^11,12,17,18^. The exact connection between differences in lipid and polar metabolite abundance observed in our study and brain function or dysfunction needs further investigation, including evaluation of differences in lipid and polar metabolite composition between various cell types of the brain, as well as research on the link between functional properties of cellular membranes and the abundance of specific lipid compounds.

Due to the nature of the samples used in our study, we were unable to distinguish between the effect of environmental and genetic factors on the inter-populational lipidome and metabolome variation. However, decoupling the genetic and environmental effects for human populations, especially in studies involving postmortem tissue samples, represents a challenge. The WE cohort examined in our study did include samples from two locations, North America and Western Europe (Table S1), but the environments at these locations are hardly distinct. Nonetheless, regardless of the cause, our study shows that the lipid and polar metabolite composition of the prefrontal cortex differs among populations, particularly in adult HC individuals.

The presence of population-specific features of the brain molecular organization has implications for further investigations, including a detailed analysis of the molecular brain composition across multiple human individuals. Additionally, our results provide a basis for the design of precision medicine studies, including clinical trial customization and treatment selection. Such studies are essential, given the multiple indications of population differences in brain morphology^17^, protein sequence variation associated with lipid abundance^52^, and differential disease susceptibility^53^.

## Methods

### Sample preparation and measurements

Prefrontal cortex samples were collected from the NICHD Brain and Tissue Bank for Developmental Disorders at the University of Maryland, the Netherlands Brain Bank, and the Chinese Brain Bank Center. Written informed consent for study participation was obtained for all samples either from the donors or their next-of-kin. According to the protocols of the NICHD Brain and Tissue Bank for Developmental Disorders at the University of Maryland, the Netherlands Brain Bank, and the Chinese Brain Bank Center, specific permission for brain autopsy and use of the brain tissue for research purposes was given by the donors or their relatives. All tissue samples were shipped by the brain banks without accompanying personal identifier information. All samples were stored at −80 °C and weighed before lipid and polar metabolite extraction, which was performed as described in^54^. Lipid measurements were conducted following the procedure by^32^, whereas polar metabolite measurement procedure is described in^54^.

### Lipid compounds preprocessing

Data alignment and pre-processing of the lipid dataset were performed using the QI software (Version 2.2, www.nonlinear.com). Lipid peaks with liquid chromatography retention times shorter than 1.5 minutes or longer than 18 minutes were excluded from the analysis. The upper mass-to-charge ratio cutoff was set to 1400 Da. This cutoff includes the vast majority of lipid classes contained in tissues, with exception of low abundance lipid classes containing four fatty acid residues, such as cardiolipins^55^. Only lipid peaks present with an intensity greater than 200 in 75% of samples were kept in the analysis. For the remaining peaks, the intensity values smaller than 200 were replaced by the constant value equal to 200. Lipid peaks with the same mass-to-charge ratio observed at numerous different retention times were excluded from the analysis as potential contaminants.

We further excluded lipid peaks potentially confounded by their processing order during mass spectrometry measurements (run order). For each lipid peak, we fitted a support vector regression (svr) model with Gaussian kernel (sklearn.svm.SVR PYTHON module, parameters: C = 100,000, epsilon 0.1, gamma 0.0001) to predict the peak intensity based on the run order, excluding three samples with the highest intensities for a more robust estimation. We then calculated the coefficient of determination (R^2^) of this prediction. The top 300 lipid peaks detected in the positive ionization mode and 250 peaks detected the negative mode with strongest run order dependency were excluded from the downstream analysis (R^2^ > 0.41 and >0.47 for positive and negative modes respectively).

The intensities of lipid compounds retained after the above-mentioned filtration procedures were upper-quartile normalized and log_2_ transformed.

### Polar compounds preprocessing

Polar metabolite data analysis was performed using the TargetSearch package according to Cuadros-Inostroza *et al*.^56^. Briefly, we describe the performed steps below. Settings for the peak-peaking and retention time alignment were as follows: m/z range from 85 up to 750 units, intensity threshold 50 units, “smoothing” peak picking method, time window 10 sec. The compound annotation was performed using the Golm metabolome database^57^, followed by the exclusion of un-annotated metabolites. Common contaminant masses 147–149 m/z were excluded, top 15 intensities from each library spectrum were selected, retention index windows were set to 2000, 1000, 200. Spearman correlation threshold = 0.95 was used for detection of correlating selective masses. The average retention time index of correlating selective masses was used for the calculation of compounds’ elution times. Multiple peaks corresponding to the same compound were collapsed based on the Spearman correlation threshold = 0.95 and elution time difference within 500 retention index units. For more details on the procedure, see^56^ and TargetSearch documentation.

The polar metabolites detected in less than 50% of the samples were excluded from the subsequent analyses. The remaining missing values were filled with the minimal intensity of the matrix. The log_2_ transformation of the polar metabolite intensities, linear regression for the experimental batch correction, and quantile normalization were applied subsequently to generate the table of polar metabolite intensities.

### Data filtration

Both polar metabolites and lipids with intensities potentially affected by postmortem interval duration (PMI) were removed. The PMI effect was determined based on the Spearman correlation between the compound intensity values across samples and samples’ PMI (nominal p-value threshold <0.01). Because we expected strong effect of age factor on lipid and metabolite abundance, only samples of individuals with ages greater than 5 years were used to estimate these correlations. The intensities of 1,670 lipids and 258 metabolites retained after above-mentioned procedure were used for all of the subsequent analysis, unless indicated otherwise (Tables S2 and S4). Additionally, part of the analysis was repeated using a more stringent exclusion criterion for potential PMI effects, where compounds were omitted from the analysis using a more relaxed p-value threshold of Spearman correlation between their intensities and PMI (nominal p-value threshold <0.1; Tables S5 and S8).

To assess the effect of sample preservation quality on the results, an additional analysis of the population differences was conducted using a subset of 82 samples with high RNA preservation (RNA integrity number (RIN) ≥7; Table S1).

### Sample sets definitions

We defined datasets DS:0-4 and DS:5-71 as follows: samples with ages less than 5 years were assigned to DS:0-4 (*n* = 74), samples with ages greater than 5 years were assigned to DS:5-71 (*n* = 229) (Table S1, Fig. 1A). We defined six age groups A1-A6 as follows: A1 included samples from less than one-year-old individuals (*n* = 24), A2 included samples from 1–4 years-old individuals (*n* = 50), A3 included samples from 5–14 years-old individuals (*n* = 41), A4 included samples from 15–24 years-old individuals (*n* = 56), A5 included samples from 25–44 years-old individuals (*n* = 56), and A6 included samples from individuals with ages greater than full 44 years of age (*n* = 76) (Table S1, Fig. 3A).

### T-distributed Stochastic neighbor embedding (t-SNE) analysis

The t-SNE analysis was conducted using “sklearn.manifold” PYTHON module with the following parameters for lipid dataset (Table S2): n_components = 2, perplexity = 30, learning rate = 100, metric = ’correlation’, early exaggeration = 100, random_state = 5, and following parameters for metabolite dataset (Table S4): n_components = 2, perplexity = 30, learning rate = 100, metric = ’correlation’, early exaggeration = 12, random_state = 0. In addition to t-SNE analysis based on all detected lipids, the same analysis was also performed based on intensities of 900 annotated lipids (Table S3).

### Total variation analysis

Percent of total variation explained by factors was estimated using analysis of variance (ANOVA) for lipid and metabolite datasets (Tables S2 and S4). The following factors were included in the analysis: age, population, RNA integrity number (RIN), postmortem interval duration (PMI), sex.

### Population specificity analysis

For both polar metabolites and lipids (Tables S2 and S4), to identify the significant intensity differences between three populations within DS:0–4 and DS:5-71 datasets, we subsampled equal number of individuals from each of the three populations within these datasets: 13 samples of each population in DS:0-4 and 25 samples of each population in DS:5-71. We then used t-test to compare the intensities in one population to the intensities in the other two populations combined. In each subsampling, compounds with t-test *p* < 0.05 after Benjamini-Hochberg correction were classified as population-specific. The subsampling procedure was performed 100 times to calculate the average number of population-specific differences for each of the three populations. To define HC-specific lipids and polar metabolites that were used in subsequent analysis, we performed the same procedure described above using the entire DS:5-71 dataset without subsampling (Tables S6 and S9).

We implemented a logistic regression model with lasso regularization to predict the population identity using DS:5-71 samples. Specifically, we randomly selected 31 samples from each population in the DS:5-71. The 93 samples selected from three populations were then randomly split into two parts. Two-thirds of the randomly selected samples (*n* = 62) were assigned as the training set, and the remaining one-third (*n* = 31) was assigned as the test set. Centering parameters (mean value of each compound) and scaling parameters (standard deviation of each compound) were estimated from the training set. Both training and test data were normalized according to these centering and scaling parameters. The logistic regression model was trained on the training set to separate one population from the other two combined using different hyperparameter C values (0.01, 0.1, 1, 10, 100, 500, 1000, 2500, 5000, 10000). Each time, the area under the receiver operating characteristic curve (ROC AUC) performance measure was calculated for the predictions of the test set. This procedure was repeated 100 times to estimate the average performance of the classifier on different test sets. Because the performance of the classifier did not depend strongly on the hyperparameter C (Fig. 2C,D), we report performances for the arbitrarily chosen C = 1000 without the risk of overfitting the model to the data used for performance validation.

We defined HC-specific compounds, both polar metabolites and lipids, using stability selection procedure, as described in^36^. Specifically, we randomly subsampled DS:5-71 individuals and split them into test and train sets, as described in the previous paragraph, followed by the construction of a HC-separating logistic regression model on the training set with lasso regularization and hyperparameter C = 1,000. Next, we identified compounds selected by the model. We performed 10,000 iterations of this procedure to rank the compounds based on the number of iterations in which they got selected by the predictive model. An arbitrary cutoff of 200 compounds for lipids and 50 compounds for polar metabolites was chosen to identify HC-specific compounds. The ranking of lipids and polar metabolites is listed in Tables S6 and S9.

### Population analysis within specified age groups

To analyze population divergence within specified age groups, we implemented classification-based and correlation-based approaches for both polar metabolite dataset and lipid dataset (Tables S2 and S4). Additionally, we selected age-unbiased population-distinguishing compounds that were not affected by the number of samples in the population and age groups. These compounds were used in the correlation-based analysis.

For the classification-based approach, we excluded the DS:0-4 samples (age groups A1 and A2) at each iteration and one sample from each of the A3-A6 age groups. Using the remaining samples, we performed stability selection and built a logistic regression using the 100 top compounds to predict the population identity of the samples excluded during the first step. We repeated this procedure until the population identity was predicted for each sample at least once and calculated the mean classification accuracy for each sample. We then used the median accuracy within a ten-sample-wide sliding window, with samples sorted according to age, to estimate the performance of the logistic regression model depending on the samples’ age.

Age-unbiased population-distinguishing compounds were defined as follows. For each population pair and each age group, four samples per population were randomly chosen 1,000 times. For each subsampling iteration, we performed t-test for each population pair and age group, and selected the compounds showing positive or negative differences with p-value < 0.1. The identified positive and negative differences were separately ranked based on the number of occurrences across the 1,000 subsampling iterations. We then selected the top-ranked 25 lipids and 10 polar metabolites showing positive differences for the corresponding population pair and age group, and same number of compounds showing negative differences. A union of these lipids and metabolites among all population comparisons and age groups was used to define the age-unbiased population-distinguishing lipid and metabolite sets.

To calculate the divergence of the three populations using correlation measurements, we randomly subsampled four samples of each population in each A1-A6 age group 10,000 times and measured, in each age group, the Spearman correlation between the means of the selected samples. The analysis was conducted using age-unbiased population-distinguishing compounds and HC-specific compounds (defined using stability selection procedure; Tables S4 and S7).

### Consistency analysis

To match the lipids from the current and the published datasets^19^, the retention times were aligned using a select set of retention times anchor points and linear interpolation between them. Lipids were matched using 5 ppm threshold and 6 seconds retention time window. Only unique matches were retained. In this section, we refer to these lipids as “matched lipid compounds” (Table S7).

To assess consistency of HC differences between current DS:5-71 and published dataset, for each dataset we calculated mean lipid intensity values of AA and WE populations samples combined and the mean lipid intensity values of HC population samples. Next, we calculated, for current and published datasets, the fold-changes of lipid intensities between HC-population and the other two by calculating differences of above-mentioned mean values. We used Spearman correlation and HC-specific lipids (Table S6) contained in the set of matched lipid compounds to calculate correlation of these fold-changes between current and published datasets.

To assess the performance of the predictive model on an external dataset, we trained a predictive model using current dataset and predicted population identity of published dataset^19^ samples, as follows. First, lipid intensities were normalized between experiments. To this purpose, 31 samples were selected from each population from DS:5-71. Mean and standard deviations were calculated for each lipid. Repeating this procedure 1,000 times, we calculated, for each lipid, an average centering (average of the mean values) and scaling (average of the standard deviation) value for the current dataset. Data was normalized according to these centering and scaling values. For published dataset, data was normalized using mean and standard deviation across samples. Because not all lipid predictors were present in published dataset and predictive power of a given lipid compound depends on the presence of other lipids in the model, we performed stability selection^36^ as described above in the Methods section, but restricting the procedure to matched lipid compounds. This produced a ranking for the matched lipids (Table S7). Next, we built logistic regression model with C = 1000 and lasso normalization to train a predictive model on DS:5-71 samples and top-ranked lipids, and predicted population identity of published dataset samples. Using a varying amount of top-ranked lipids, we observed that the performance of the model peaked at 22 predictors (Fig. S8).

### Lipid annotation and enrichment analysis

Lipid annotation was performed using mass search with a tolerance of 5 ppm against the LIPID MAPS database^34^ (Table S3). The possible adducts were set to [M + H]^+^, [M + Na]^+^, [M + NH4]^+^ in positive ionization mode, and [M-H]^−^, [M-H + HCOOH]^−^, [M-H + CH3COOH]^−^ in negative ionization mode. For functional enrichment analysis, Kyoto Encyclopedia of Genes and Genomes (KEGG) database^37^ was used to link lipids and polar metabolites to genes. We used hypergeometric test to assess, for each metabolic pathway, the enrichment of genes linked to HC-specific lipids and metabolites, compared to genes linked to all annotated lipids and metabolites (Tables S3 and S4). HC-specific lipids and metabolites defined from stability selection (Tables S6 and S9) were used in this analysis.

## Supplementary information

SUPPLEMENTARY INFORMATION

S1

S2

S3

S4

S5

S6

S7

S8

S9

S10

## Data Availability

The datasets used and analyzed during the current study are available from the corresponding author upon reasonable request.
