# Supplementary material for: Differences in lipidome and metabolome organization of prefrontal cortex among human populations
Source: Sci Rep. 2019 Dec 4;9:18348. doi: 10.1038/s41598-019-53762-6 (PMC6893025; doi:10.1038/s41598-019-53762-6)
Supplement: Supplementary file 1 — SUPPLEMENTARY INFORMATION [file 41598_2019_53762_MOESM1_ESM.pdf]

SUPPLEMENTARY INFORMATION:

Differences in lipidome and metabolome organization of prefrontal cortex among human populations

Anna Tkachev[1,2,§], Vita Stepanova[1,2,§], Zhang Lei[3,§], Ekaterina Khrameeva[1], Dmitry Zubkov[1], Patrick Giavalisco[4,*], Philipp Khaitovich[1,3,5,*]

[1] Skolkovo Institute of Science and Technology, 143028 Moscow, Russia.

[2] Institute for Information Transmission Problems, Russian Academy of Sciences, Bolshoy Karetny Per. 19/1, 127051 Moscow, Russia.

[3] CAS Key Laboratory of Computational Biology, CAS-MPG Partner Institute for Computational Biology, 320 Yue Yang Road, 200031 Shanghai, China.

[4] Max Planck Institute for Biology of Aging, Joseph-Stelzmann-Straße 9B, 50931 Köln, Germany

[5] Max Planck Institute for Evolutionary Anthropology, Deutscher Platz 6, 04103 Leipzig, Germany.

[§] Contributed equally

[*] Correspondence to: patrick.giavalisco@age.mpg.de and khaitovich@eva.mpg.de

Supplementary Tables

Supplementary Table S1. List of samples used for lipidomic and metabolic measurements.

Supplementary Table S2. Abundance of 1,670 confounder-free distinct lipid peaks.

Supplementary Table S3. Computational annotation of lipid dataset based on LIPID MAPS database.

Supplementary Table S4. Abundance of 258 confounder-free metabolites.

Supplementary Table S5. Abundance of 1,419 lipids remaining after exclusion of compounds showing even weak correlation (nominal p-value threshold < 0.1) between their abundance and PMI values.

Supplementary Table S6. Ranking of lipids based on stability selection, including list of HC-specific lipids identified using t-test.

Supplementary Table S7. Lipids matching between current and published datasets.

Supplementary Table S8. Abundance of 192 metabolites remaining after exclusion of compounds showing even weak correlation (nominal p-value threshold < 0.1) between their abundance and PMI values.

Supplementary Table S9. Ranking of polar metabolites based on stability selection, including list of HC-specific metabolites identified using t-test.

Supplementary Table S10. KEGG pathway enrichment analysis based on HC-specific lipids and polar metabolites.

Supplementary Figure

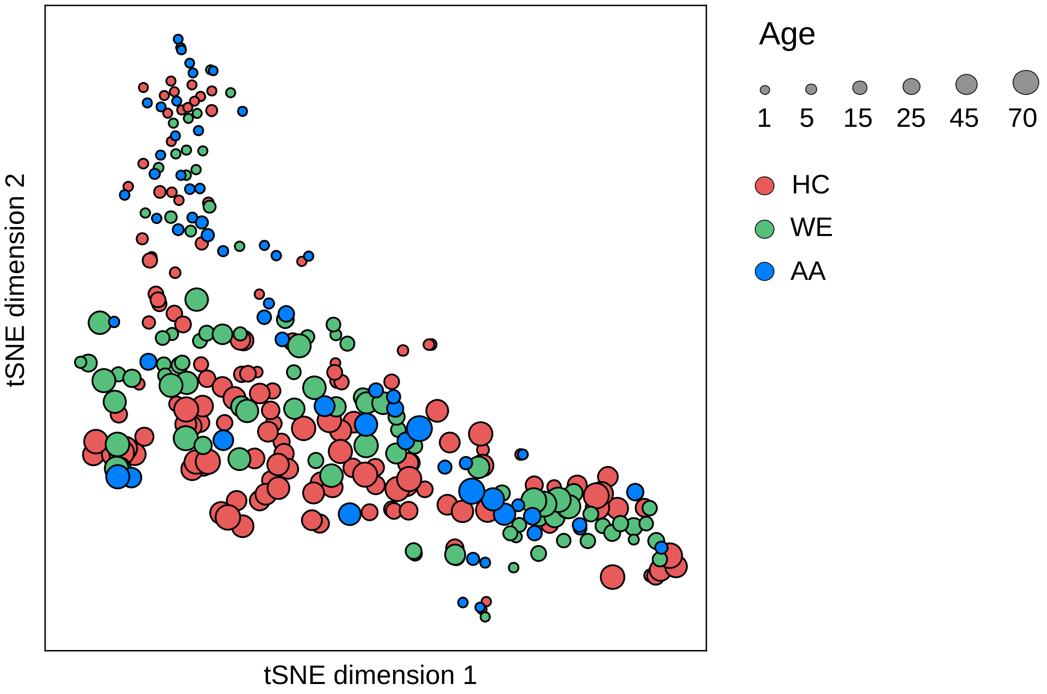

Supplementary Figure S1. Lipid abundance variation visualized using computationally annotated lipids.

T-distributed stochastic neighbor embedding (t-SNE) visualizing abundance variation of 900 computationally annotated lipids among individuals. Each circle represents an individual. The sizes of the circles are related to the individuals' age – larger circles represent older individuals. Colors represent populations, as described by the in-figure legend.

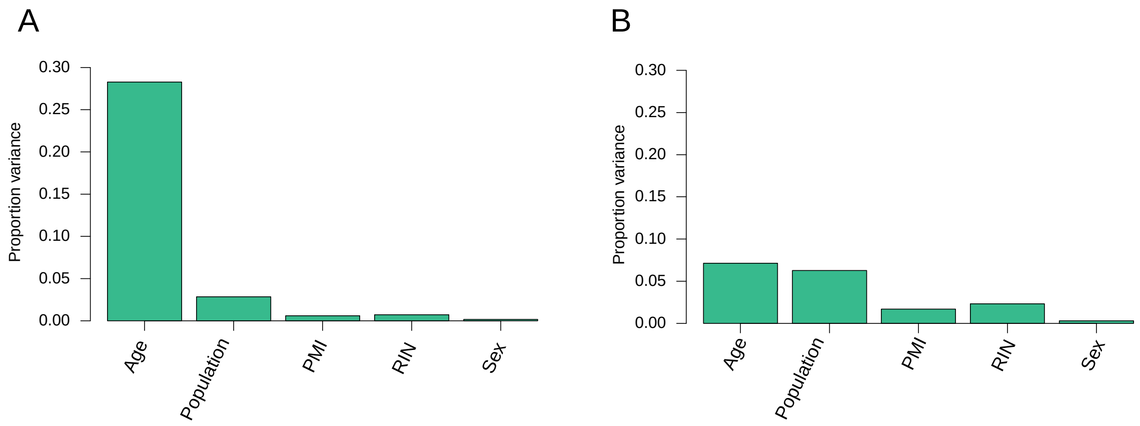

Supplementary Figure S2. Variation analysis of lipid and metabolite intensities.

Proportion of the total variance explained by the given factor, calculated using ANOVA. PMI – postmortem interval duration, RIN – RNA integrity number. (A) lipid dataset, (B) polar metabolite dataset.

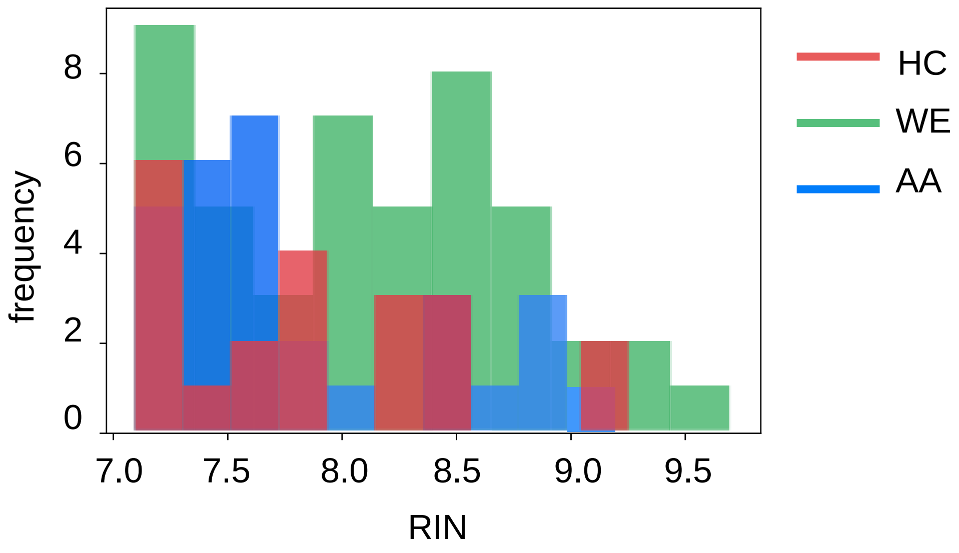

Supplementary Figure S3. Distribution of RNA integrity number (RIN) values in subset of DS:5-71 samples with good tissue preservation (RIN ≥ 7).

RIN distribution in DS:5-71 samples retained after RIN ≥ 7 RNA quality cutoff. Colors correspond to populations: red – Han Chinese (HC), green – Western Europeans (WE), blue – African Americans (AA).

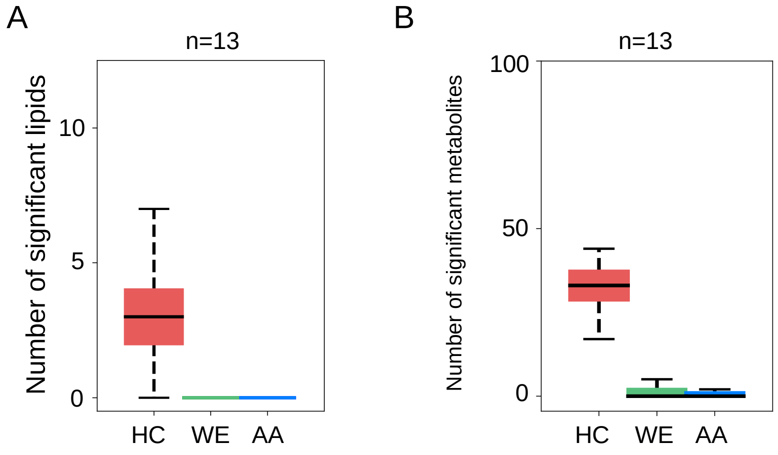

Supplementary Figure S4. Lipidome and metabolome population-specific differences calculated using DS:5-71 samples with good tissue preservation (RNA integrity number RIN ≥ 7).

Number of lipids (A) and polar metabolites (B) with significant abundance differences between one population and the other two combined, estimated by subsampling 13 samples with RIN ≥7 from each of the three populations 100 times. The colors represent populations: red – Han Chinese (HC), green – Western Europeans (WE), blue – African Americans (AA).

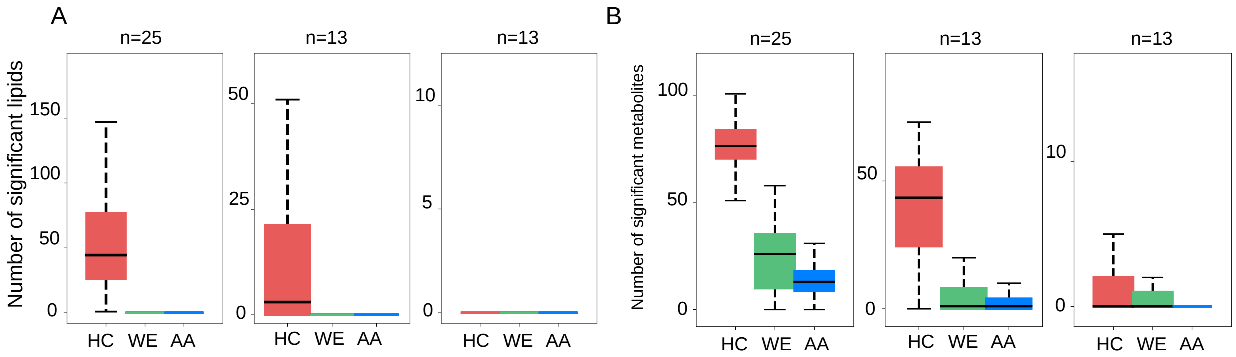

Supplementary Figure S5. Lipidome and metabolome population-specific differences after exclusion of compounds showing possible correlation between their abundance and postmortem interval duration (PMI).

Number of lipids (A) and metabolites (B) with significant abundance differences between one population and the other two combined, estimated by subsampling n individuals from each of the three populations 100 times. The numbers of subsampled individuals n used in analysis are marked on top of the panels. The results are shown for DS:5-71 (left, n = 25), DS:5-71 with same number n as DS:0-4 (middle, n = 13), and DS:0-4 (right, n = 13). The colors represent populations: red – Han Chinese (HC), green – Western Europeans (WE), blue – African Americans (AA). Compounds showing correlation (nominal p-value threshold < 0.1) between their abundance and PMI duration values were excluded from this analysis.

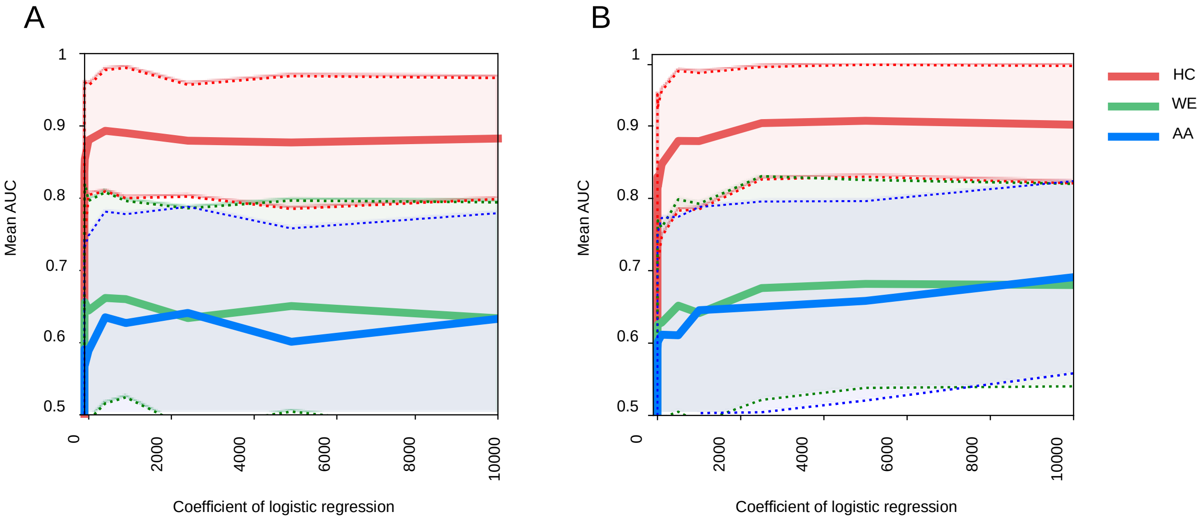

Supplementary Figure S6. Lasso logistic regression performance estimates based on DS:5-71 samples with good tissue preservation (RNA integrity number RIN ≥ 7).

The Area Under the ROC Curve (AUC) estimates for the lasso logistic regression models separating samples from one population and samples from the other two combined, calculated using different values of hyperparameter C (inverse of regularization strength). The models were based on the lipid (A) and polar metabolite (B) abundance in DS:5-71 samples with good tissue preservation (RIN ≥ 7). Lines correspond to the means of AUC values estimated on different test sets. The shaded areas indicate the standard deviations of AUC values estimated on different test sets. The colors correspond to populations, as described by in-figure legend.

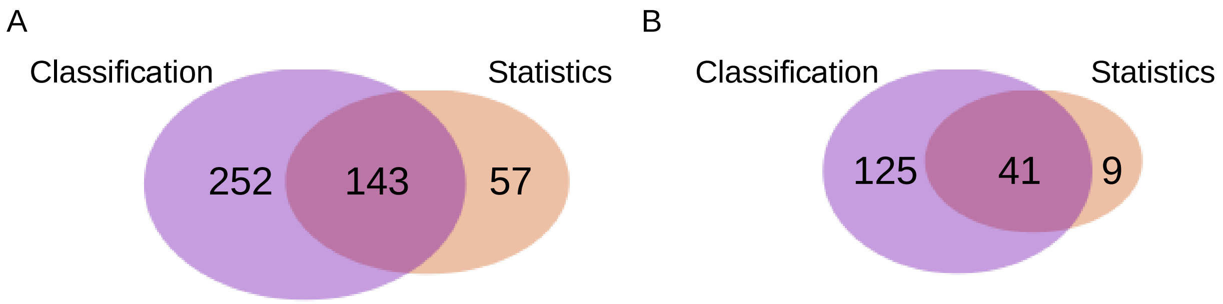

Supplementary Figure S7. Intersection of HC-specific lipids and polar metabolites defined using stability selection procedure or t-test statistics.

Intersection of HC-specific lipids (A) and polar metabolites (B) identified using stability selection procedure (purple) or t-test statistics (orange).

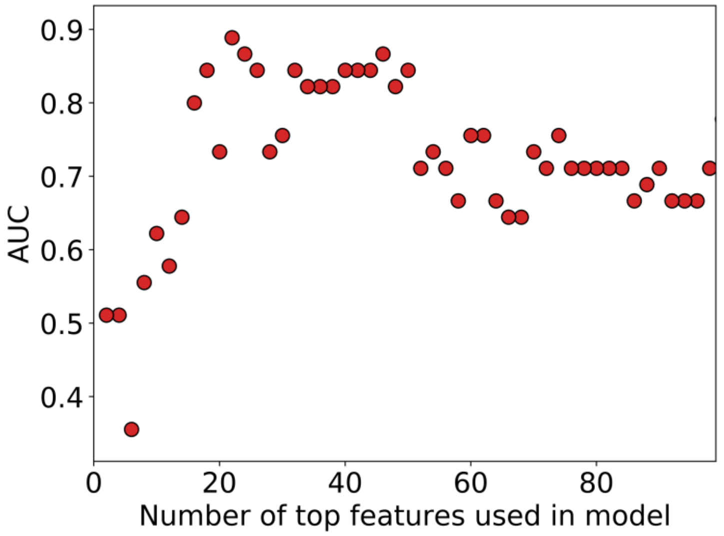

Supplementary Figure S8. Performance of lasso logistic regression on published dataset.

The Area Under the ROC Curve (AUC) estimates for the lasso logistic regression model trained on current DS:5-71 and validated on published external dataset (19). Model was built using varying number of top-ranked lipids defined using stability selection. X-axis shows number of top-ranked lipids used in the model.

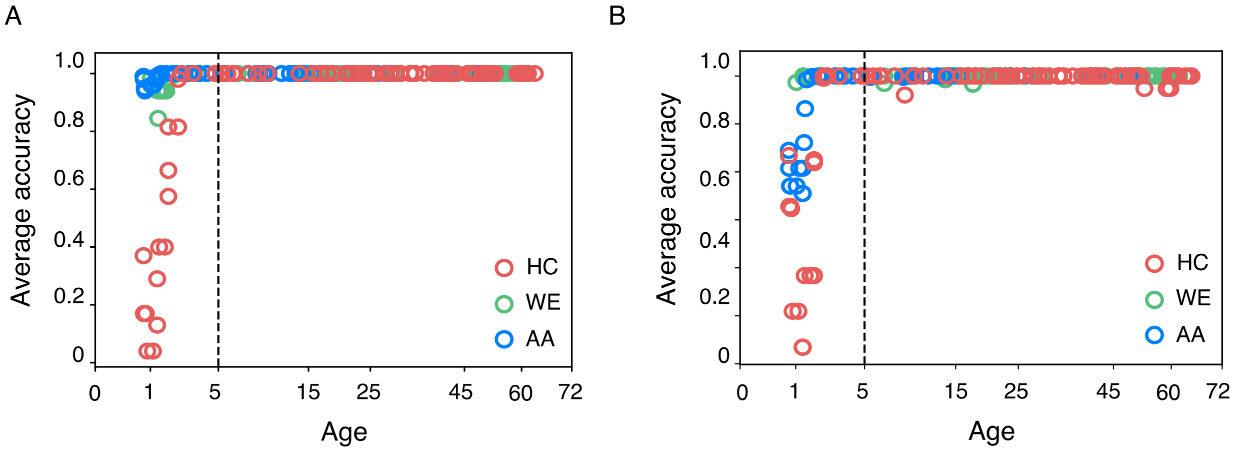

Supplementary Figure S9. The relationship between accuracy of lasso logistic regression separating HC samples from the WE and AA samples, and individuals' age.

Classification accuracy of samples depending on individuals' age. X-axis shows age of individuals in years. In each iteration, all samples from A1 and A2 age groups were excluded from the train set, as well as one sample of each population from each of the A3-A6 age groups. Lasso logistic regression was built to separate HC samples from AA and WE samples, and performance was calculated for the samples excluded from the training set. Subsequently, mean accuracies for each sample were calculated. The median accuracy within a ten-sample-wide sliding window, with samples sorted according to age, was used to estimate the model accuracy shown on the plot.

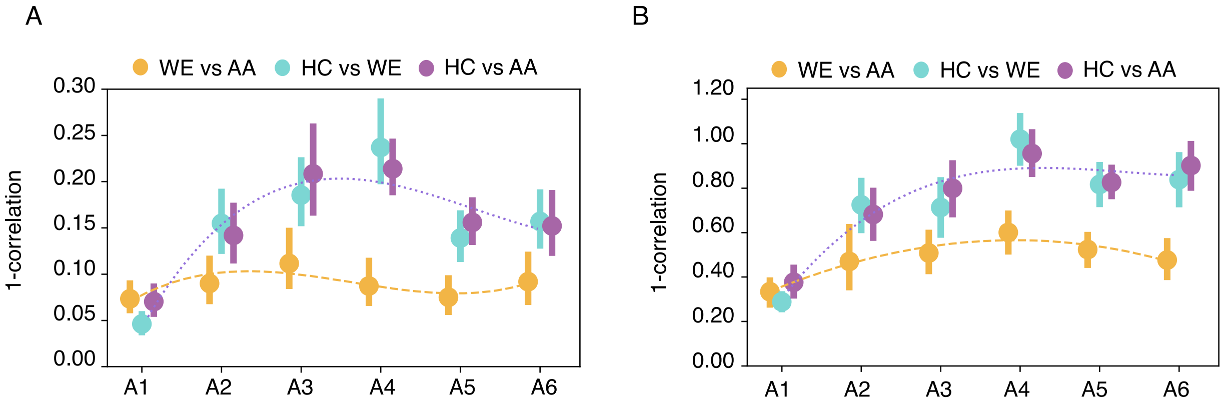

Supplementary Figure S10. Pairwise population differences across lifespan estimated using HC-specific lipids and polar metabolites.

Pairwise population differences estimated based on the abundance of HC-specific lipids (B) and polar metabolites (C) identified using stability selection procedure. Differences were calculated in each age group A1-A6 using correlations of population-mean abundances based on four samples subsampled from each population. Y-axis represents distance values calculated as one minus these correlation values. Circles represent the median distance values estimated by subsampling within each age group 10,000 times. Vertical lines extend to the upper and lower quartile values in each age group. The dotted blue line represents a smooth spline curve fitted to the average of the HC-WE and HC-AA distances. The dashed orange line represents a smooth spline curve fitted to the WE-AA distance.
